# Simple Syntheses of Two New Benzo-Fused Macrocycles Incorporating Chalcone Moiety

**DOI:** 10.1155/2014/485014

**Published:** 2014-10-29

**Authors:** Rina Mondal, Swati Samanta, Saheli Sarkar, Asok K. Mallik

**Affiliations:** Department of Chemistry, Jadavpur University, Kolkata 700 032, India

## Abstract

Simple syntheses of the benzo-fused 26-membered macrocyclic bischalcone (19*E*,43*E*)-2.11.27.36-tetroxaheptacyclo[44.4.0.0^4,9^.0^12,17^.0^21,26^.0^29,34^.0^37,42^]pentaconta-1(46),4(9),5,7,12(17),13,15,19,21,23,25,29,31,33,37,39,41,43,47,49-icosaene-18,45-dione (**3**) and the benzo-fused 13-membered macrocyclic chalcone (19*E*)-2.11-dioxatetracyclo[19.4.0.0^4,9^.0^12,17^]pentacosa-1(25),4(9),5,7,12(17),13,15,19,21,23-decaen-18-one (**5**) using very common starting materials and reagents are described. The compounds are new and they have been characterized from their analytical and spectral data.

## 1. Introduction 

Chalcones (1,3-diphenyl-2-propen-1-ones) [1] are known to possess a range of important biological activities, such as antibacterial [2], antifungal [3], antileishmanial [4], antimalarial [4], antifilarial [5] anti-inflammatory [6–8], antiprotozoal (antileishmanial and antipanosomal) [9], antimicrobial [10–13], larvicidal [14], anticonvulsant [15], anti-HIV [16], antitumor [17], and anticancer [18] activities, and they could be readily transformed into varieties of other compounds, many of which are biologically active heterocycles [19, 20]. Owing to such biological activities of chalcones, the chemical literature shows the synthesis of a wide range of chalcones and their analogues [4, 8–13, 21–23]. Again, since macrocyclic compounds are well-known for their ability to show the important property of molecular recognition, macrocyclic systems containing the chalcone moiety are expected to generate compounds having interesting biological and material properties [24, 25]. Our research on such compounds has been initiated through the synthesis of a number of macrocyclic bis- and monochalcones reported recently [26]. In continuation of that study we have synthesized a benzo-fused 26-membered macrocyclic bischalcone, namely, (19*E*,43*E*)-2.11.27.36-tetroxaheptacyclo [44.4.0.0^4,9^.0^12,17^.0^21,26^.0^29,34^.0^37,42^] pentaconta-1(46),4(9),5,7,12(17),13,15,19,21,23,25,29,31,33,37,39,41,43,47,49-icosaene-18,45-dione (**3**), and a benzo-fused 13-membered macrocyclic monochalcone, namely, (19*E*)-2.11-dioxatetracyclo[19.4.0.0^4,9^.0^12,17^] pentacosa-1(25),4(9),5,7,12(17),13,15,19,21,23-decaen-18-one (**5**), by use of readily available starting materials. Herein we report the synthesis of these two hitherto unknown compounds.

## 2. Results and Discussion

**Scheme 1:**
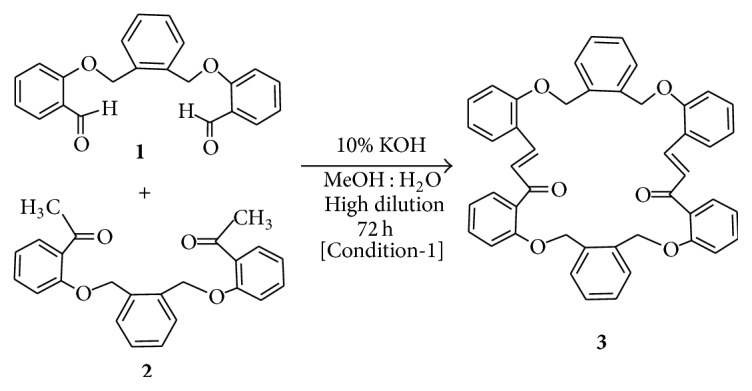
Synthesis of the macrocyclic bischalcone** 3**.

**Scheme 2:**
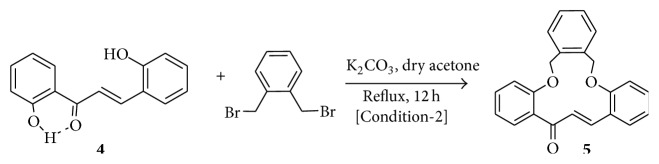
Synthesis of the macrocyclic monochalcone** 5**.

**Scheme 3:**
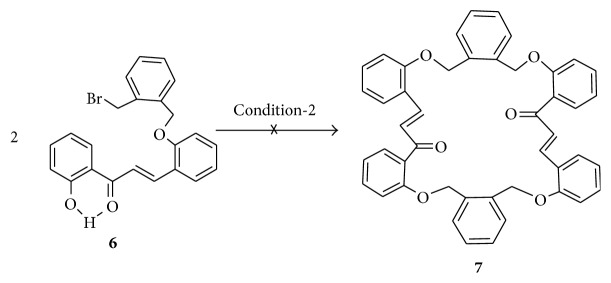
A possible way of formation of macrocyclic bischalcone from** 6**.

Alkylation products of salicylaldehyde and* o*-hydroxyacetophenone by the use of 1,2-(bis-bromobenzyl)benzene as alkylating agent were first prepared. The resulting dialdehyde (**1**) and diketone (**2**), both new compounds, were then subjected to Claisen-Schmidt reaction under high dilution condition when the macrocyclic bischalcone** 3 **was obtained in moderate yield (Scheme 1). In order to achieve the synthesis of** 5**, 2,2′-dihydroxychalcone (**4**) was first constructed from* o*-hydroxyacetophenone and salicylaldehyde by Claisen-Schmidt reaction. The reaction between** 4** and 1,2-(bis-bromobenzyl)benzene was then done by following the typical procedure for alkylation of phenols (K_2_CO_3_/acetone, reflux, 12 h) (Scheme 2). This reaction gave** 5** in very good yield and no trace of any macrocyclic bischalcone** 7** (a possible product through bimolecular cyclization of the intermediate** 6**) (Scheme 3).

The new compounds** 3** and** 5** were characterized from their analytical and spectral data which are presented in the Experimental Section as well as in the Supplementary File in the Supplementary Material available online at http://dx.doi.org/10.1155/2014/485014.

## 3. Experimental 

Melting points were taken in open capillary tubes and are uncorrected. IR spectra were recorded on a Perkin Elmer FT-IR spectrophotometer (Spectrum BX II) in KBr pellets. ^1^H and ^13^C NMR spectra were recorded in CDCl_3_ on a Bruker AV-300 (300 MHz) spectrometer. Analytical samples were routinely dried* in vacuo* at room temperature. Microanalytical data were recorded on a Perkin-Elmer 2400 Series II C, H, N analyzer. ESIMS(+) mass spectrum of** 3** was measured with a Waters Micromass Q-Tof micro instrument. Column chromatography was performed with silica gel (100–200 mesh) and TLC with silica gel G made of SRL Pvt. Ltd. Petroleum ether used had the boiling range of 60–80°C. ^1^H and ^13^C NMR and mass spectra of different compounds can be found in the Supplementary Material.

### 3.1. 1,2-Bis(bromomethyl)benzene

This compound was prepared from* o*-xylene by benzylic bromination with NBS [NBS (2.2 equiv.), (PhCOO)_2_ (trace), refluxed in CCl_4_, 12 h, yield: 83%), m.p. 94°C (lit. [27] 92–96°C); ^1^H NMR (CDCl_3_): *δ* 4.66 (br. s, 4H, –CH_2_-Br), 7.29–7.31 (m, 2H, Ar-H), 7.33–7.38 (m, 2H, Ar-H).

### 3.2. 1,2-Bis(2-formylphenoxymethyl)benzene (**1**)

A mixture of salicylaldehyde (2 mmol) and 1,2-bis(bromomethyl)benzene (1 mmol) was refluxed in methanolic KOH (5%, 25 mL) for 7 h. Removal of methanol by distillation and addition of water followed by extraction with ethyl acetate gave crude alkylation product** 1**, which was purified by rapid column chromatography followed by crystallization from CHCl_3_-petroleum ether (yield: 66%). The physical and spectral data of** 1** were as follows: colourless needles, m.p. 118–120°C; ^1^H NMR (300 MHz, CDCl_3_): *δ* 5.31 (br. s, 4H, 2 × –OCH_2_–), 7.05 (dt, 4H,* J* = 7.2 and 1.5 Hz), 7.42–7.45 (m, 2H, ArH), 7.50–7.57 (m, 4H), 7.82 (dd, 2H,* J *= 7.8 and 1.8 Hz), and 10.45 (br. s, 2H, 2 × –CHO).

### 3.3. 1,2-Bis(2-acetylphenoxymethyl)benzene (**2**)

A mixture of* o*-hydroxyacetophenone (2 mmol), 1,2-bis(bromomethyl)benzene (1 mmol) and anhydrous K_2_CO_3_ (3 g.) was refluxed in dry acetone for 12 h. Usual work-up followed by purification of the resulting crude material by column chromatography over silica gel afforded pure** 2 **(yield: 78%). The physical and spectral data of** 2** were as follows: colourless needles (CHCl_3_-petroleum ether), m.p. 74°C; ^1^H NMR (300 MHz, CDCl_3_): *δ* 2.53 (s, 6H, 2 × –COCH_3_), 5.27 (br. s, 4H, –OCH_2_), 7.03 (dt, 4H,* J *= 8.4 and 1.8 Hz), 7.40–7.46 (m, 4H), 7.54 (dt, 2H,* J *= 8.4 and 1.8 Hz), and 7.71 (dd, 2H,* J* = 7.8 and 1.8 Hz).

### 3.4. (19*E*,43*E*)-2.11.27.36-Tetroxaheptacyclo[44.4.0.0^4,9^.0^12,17^.0^21,26^.0^29,34^.0^37,42^]pentaconta-1(46),4(9),5,7,12(17),13,15,19,21,23,25,29,31,33,37, 39,41,43,47,49-icosaene-18,45-dione (**3**)

A mixture of the dialdehyde** 1** (1 mmol) and the diketone** 2** (1 mmol) was dissolved in a KOH solution (10%, 75 mL) in MeOH-H_2_O (3 : 1) and the mixture was stirred at room temperature. A precipitate began to be formed after* ca.* 5 h of stirring. The stirring was continued for 72 h and then the solid was collected. The solid thus obtained was almost pure and it was further purified by column chromatography over silica gel followed by crystallization from CHCl_3_-petroleum ether (yield: 49%). The analytical and spectral data of the macrocyclic product** 3** were as follows: light yellow cubes; m.p. 205–207°C; IR (KBr, cm^−1^): 1598 (C=O), 1567, 1485, 1446, 1384 cm^−1^; ^1^H NMR (300 MHz, CDCl_3_): *δ* 4.85 (s, 4H, –O–CH_2_–), 5.06 (s, 4H, –CH_2_–O–), 6.77 (d, 4H,* J* = 7.2 Hz), 6.80 (t, 2H,* J* = 7.5 Hz), 6.97 (t, 4H,* J* = 7.8 Hz), 7.18–7.26 (m, 10H), 7.27 (d, 2H,* J* = 16.2 Hz, 2 × H-*α*), 7.38 (dt, 2H,* J* = 7.5 and 1.5 Hz), 7.47 (dd, 2H,* J *= 8.4 and 1.5 Hz), 7.70 (d, 2H,* J* = 16.2 Hz, 2 × H-*β*); ^13^C NMR (75 MHz, CDCl_3_): *δ* 68.23, 68.48, 112.47, 112.75, 121.04, 121.21, 123.98, 127.65, 128.10, 128.11, 128.48, 129.40, 129.60, 130.10, 130.21, 131.47, 132.23, 133.81, 134.42, 140.17, 156.42, 157.10, 194.64 (C=O); MS (TOF MS ES^+^):* m/z* 707.18 (M+Na)^+^, 685.19 (M+H)^+^. Elemental analysis: Calcd. for C_46_H_36_O_6_:C, 80.68; H, 5.30. Found: C, 80.56; H 5.42%.

### 3.5. 2,2′-Dihydroxychalcone (**4**)

This chalcone was prepared by condensation of* o*-hydroxyacetophenone and salicylaldehyde with aqueous alkali, m.p. 162–164°C (lit. [28] 168°C).^ 1^H NMR (300 MHz, CDCl_3_): *δ* 5.74 (br. s, 1H, 2-OH), 6.88 (br. d, 1H,* J* = 8.1 Hz), 6.94–7.07 (m, 3H), 7.32 (dt, 1H,* J* = 7.8 and 1.8 Hz), 7.52 (dt, 1H,* J* = 7.5 and 1.5 Hz), 7.63 (dd, 1H,* J* = 7.8 and 1.9 Hz), 7.87 (d, 1H,* J* = 15.6 Hz, H-*α*), 7.96 (dd, 1H,* J* = 8.1 and 1.5 Hz), 8.21 (d, 1H,* J* = 15.6 Hz, H-*β*), 12.92 (s, 1H, 2′-OH).

### 3.6. (19*E*)-2.11-Dioxatetracyclo[19.4.0.0^4,9^.0^12,17^]pentacosa-1(25),4(9),5,7,12(17),13,15,19,21,23-decaen-18-one (**5**)

To a mixture of** 4** (1 mmol) and 1,2-bis(bromomethyl)benzene (1 mmol) in dry acetone (25 mL), anhydrous K_2_CO_3_ (3 g) was added and the mixture was refluxed with stirring for 12 h. Usual work-up of the reaction mixture followed by chromatography of the crude product over silica gel using petroleum ether-ethyl acetate (90 : 10, v/v) gave pure** 5** as light yellow crystals (yield: 57%), m.p. 160–162°C, IR (KBr) cm^−1^: 3028, 2903, 1585, 1562, 1470, 1453, 1436, 1297, 1249, 1152, 1058, 960, 745. ^1^H NMR (300 MHz, CDCl_3_): *δ* 5.28 (s, 4H, 2 × ArCH_2_O–), 7.01–7.10 (m, 2H), 7.11 (d, 1H,* J *= 16.5 Hz, H-*α*), 7.23–7.38 (m, 7H), 7.45–7.51 (m, 2H), 7.58 (dd, 1H,* J *= 7.5 and 1.8 Hz, proton* ortho* to C=O), 7.66 (d, 1H,* J *= 16.5 Hz, H-*β*), ^13^C NMR (75 MHz, CDCl_3_): *δ* 68.65, 71.70, 113.51, 119.01, 121.67, 123.46, 128.43, 128.81, 129.19, 129.77, 129.79, 129.87, 130.68, 130.88, 131.89, 132.62, 135.18, 135.29, 141.56, 156.71, 156.97, 195.23. MS (TOF MS ES^+^):* m/z* (M+Na)^+^ 365.00. Elemental analysis: Calcd. for C_23_H_18_O_3_: C, 80.68; H, 5.30. Found: C, 80.45; H, 5.44%.

## 4. Conclusion

Thus, we report very simple syntheses of two new benzo-fused macrocycles incorporating chalcone moiety by the use of very common starting materials. The compounds may find important applications.

## Supplementary Material

Supplementary Material: Two tables containing the analytical and spectral data of 3 and 5 and ten figures showing the spectra of different compounds have been given.
