# Supplementary material for: Simple Syntheses of Two New Benzo-Fused Macrocycles Incorporating Chalcone Moiety
Source: Int Sch Res Notices. 2014 Oct 29;2014:485014. doi: 10.1155/2014/485014 (PMC4897219; doi:10.1155/2014/485014)

Supplementary File

Simple Syntheses of Two New Benzo-fused Macrocycles Incorporating Chalcone Moiety

Rina Mondal, Swati Samanta, Saheli Sarkar, and Asok K. Mallik*

Department of Chemistry, Jadavpur University, Kolkata-700 032, India

(19*E*,43*E*)-2.11.27.36-Tetroxaheptacyclo[44.4.0.0^{4,9}.0^{12,17}.0^{21,26}.0^{29,34}.0^{37,42}]pentaconta-1(46),4(9),5,7,12(17),13,15,19,21,23,25,29,31,33,37,39,41,43,47,49-icosaene-18,45-dione (3)

Molecular formula	$C_{46}H_{36}O_6$ (684.77)
Physical state	Light yellow cubes
M. p.	205-207 °C
Elemental analysis	Calcd C, 80.68; H, 5.30%<br>Found C, 80.56; H 5.42%
IR spectrum (KBr)	1598 (C=O), 1567, 1485, 1446, 1384 cm^{-1}
^{1}H NMR spectrum<br>(300 MHz, $CDCl_3$)	δ_H = 4.85 (s, 4H, -O-C<u>H</u>$_2$-), 5.06 (s, 4H, -CH_2-O-), 6.77 (d, 4H, *J* = 7.2 Hz), 6.80 (t, 2H, *J* = 7.5 Hz), 6.97 (t, 4H, *J* = 7.8 Hz), 7.18-7.26 (m, 10H), 7.27 (d, 2H, *J* = 16.2 Hz, 2 × H-α), 7.38 (dt, 2H, *J* = 7.5 and 1.5 Hz), 7.47 (dd, 2H, *J* = 8.4 and 1.5 Hz), 7.70 (d, 2H, *J* = 16.2Hz, 2 × H-β) **[Fig. 4]**
^{13}C NMR spectrum<br>(75 MHz, $CDCl_3$)	δ_C = 68.23, 68.48, 112.47, 112.75, 121.04, 121.21, 123.98, 127.65, 128.10, 128.11, 128.48, 129.40, 129.60, 130.10, 130.21, 131.47, 132.23, 133.81, 134.42, 140.17, 156.42, 157.10, 194.64 (C=O) **[Fig. 5]**
MS (TOF MS ES$^+$):	*m/z* 707.18 (M+Na)$^+$, 685.19 (M+H)$^+$ **[Fig. 6]**

(19*E*)-2.11-dioxatetracyclo[19.4.0.0^{4,9}.0^{12,17}]pentacosa-1(25),4(9),5,7,12(17),13,15,19,21,23-decaen-18-one (5)

Molecular formula	$C_{23}H_{18}O_3$(342.12)
Physical state	Light yellow cubes
M. p.	160-162 °C
Elemental analysis	Calcd.: C, 80.68; H, 5.30.<br><br>Found: C, 80.45; H, 5.44%.
IR spectrum (KBr)	3028, 2903, 1585, 1562, 1470, 1453, 1436, 1297, 1249, 1152, 1058, 960, 745 cm^{-1}
^{1}H NMR spectrum<br><br>(300 MHz, $CDCl_3$)	δ_H: 5.28 (s, 4H, 2 × $ArCH_2O$-), 7.01-7.10 (m, 2H), 7.11 (d, 1H, *J* = 16.5 Hz, H-α), 7.23–7.38 (m, 7H), 7.45–7.51 (m, 2H), 7.58 (dd, 1H, *J* = 7.5 and 1.8 Hz, proton *ortho* to C=O), 7.66 (d, 1H, *J* = 16.5 Hz, H-β) **[Fig. 8]**.
^{13}C NMR spectrum<br><br>(75 MHz, $CDCl_3$)	δ_C: 68.65, 71.70, 113.51, 119.01, 121.67, 123.46, 128.43, 128.81, 129.19, 129.77, 129.87, 130.68, 130.88, 131.89, 132.62, 135.18, 135.29, 141.56, 156.71, 156.97, 195.23**[Fig. 9]**.
MS (TOF MS ES^{+}):	*m/z* 365 $(M+Na)^{+}$ **[Fig. 10].**

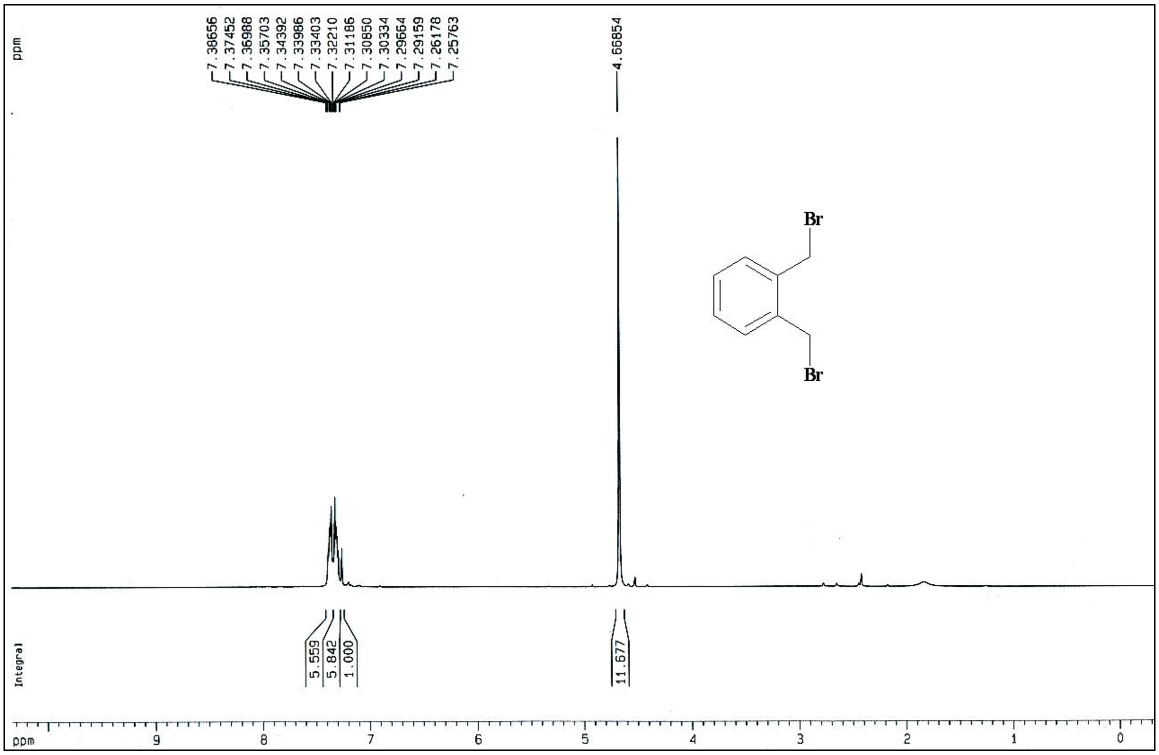

Fig. 1: ^{1}H NMR Spectrum of 1,2-Bis(bromomethyl)benzene (300 MHz, $CDCl_3$)

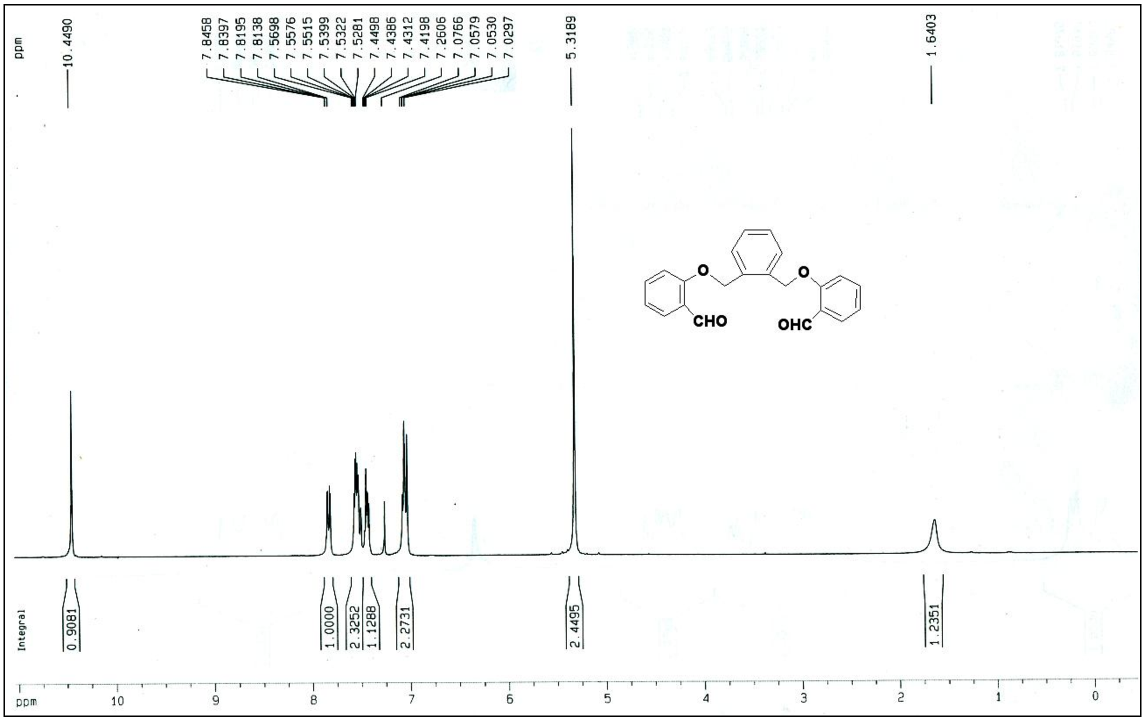

Fig. 2: ^{1}H NMR Spectrum of 1 (300 MHz, $CDCl_3$)

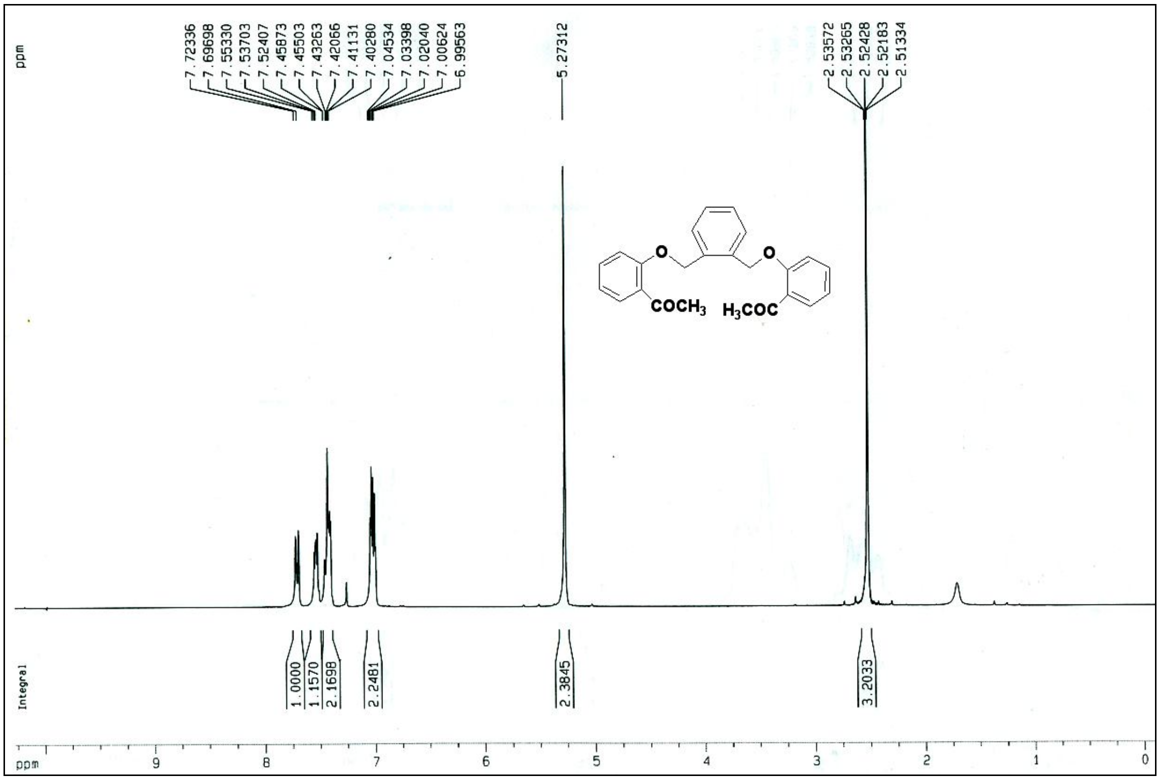

Fig. 3: ^{1}H NMR Spectrum of 2 (300 MHz, $CDCl_3$)

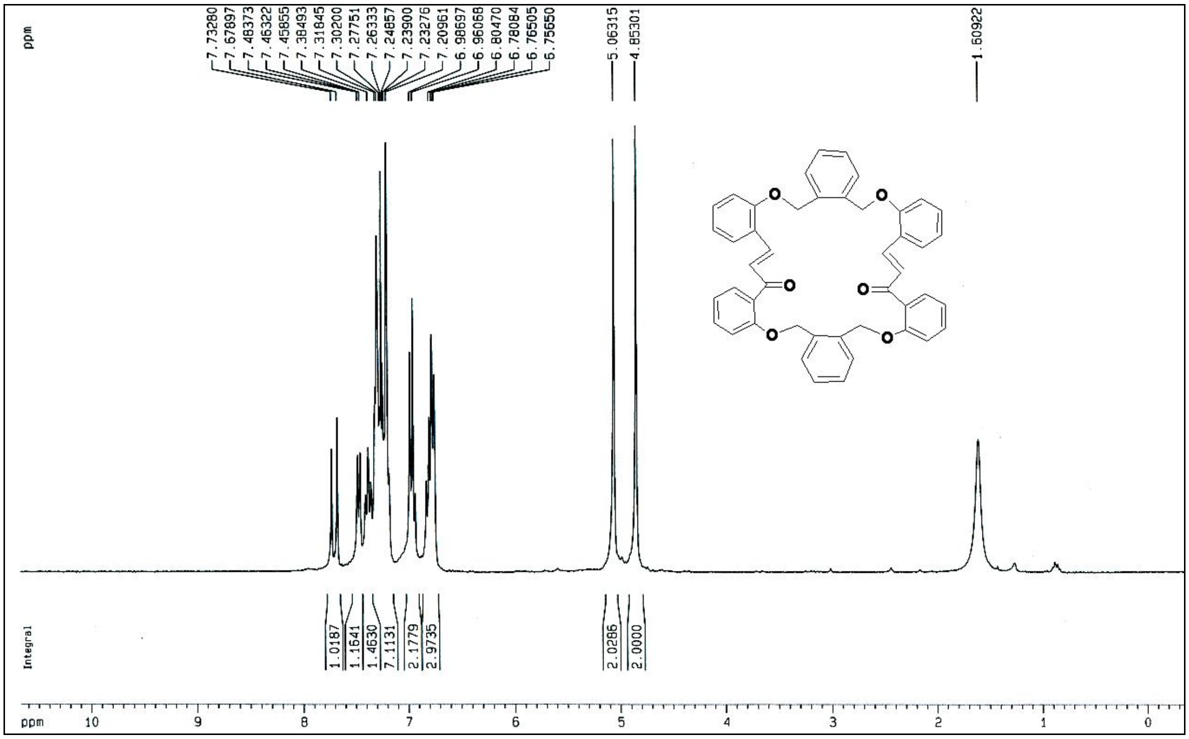

Fig. 4: ^{1}H NMR Spectrum of 3 (300 MHz, $CDCl_3$)

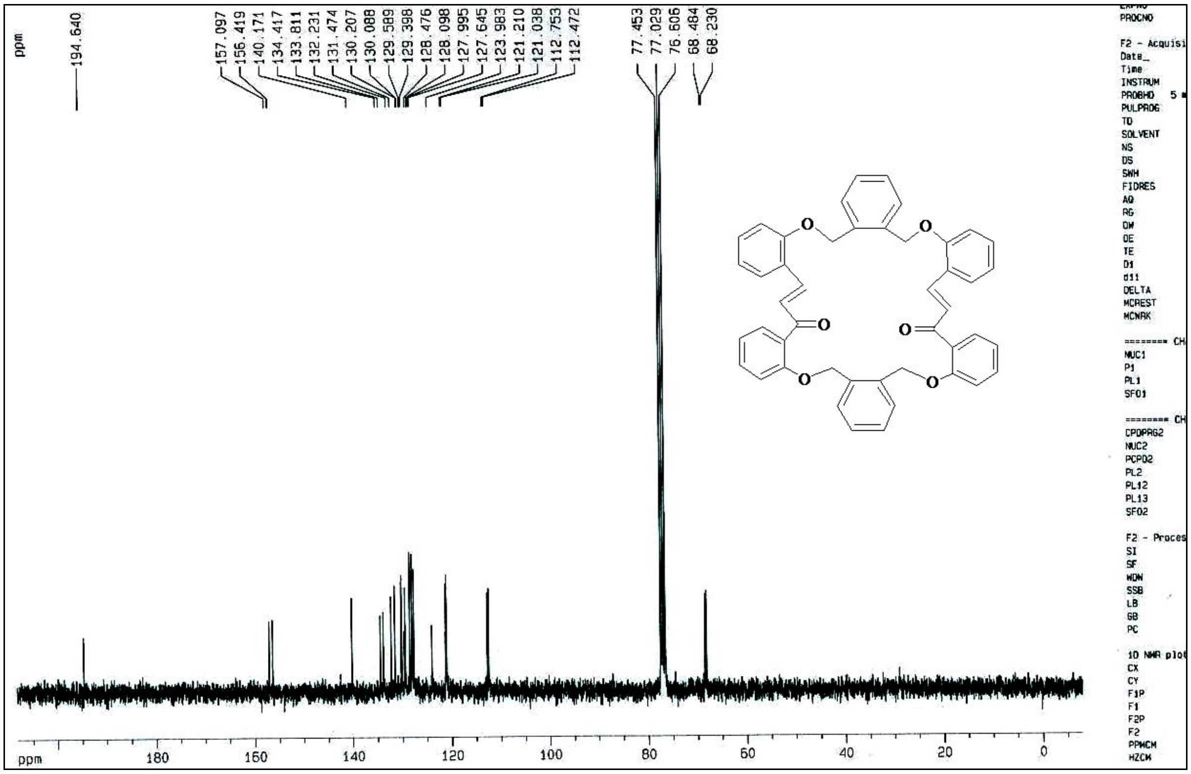

Fig. 5: ^{13}C NMR Spectrum of 3 (75 MHz, $CDCl_3$)

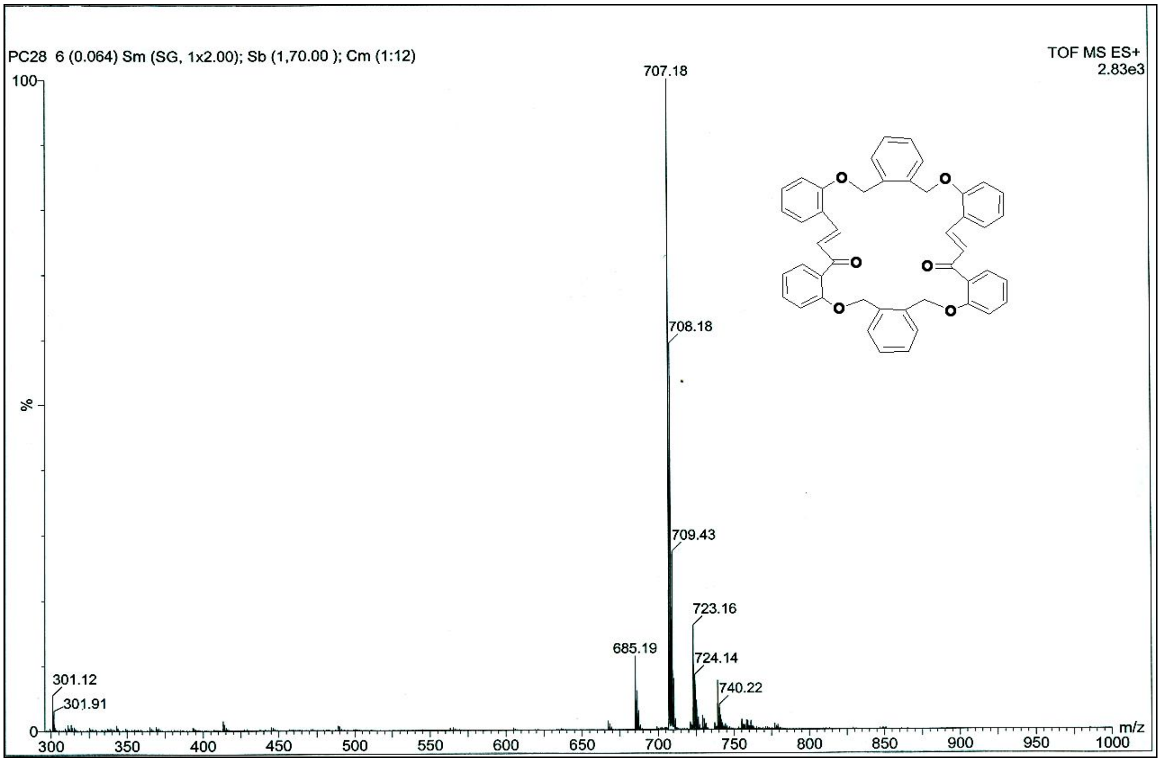

Fig. 6: TOF MS ES+ Spectrum of 3

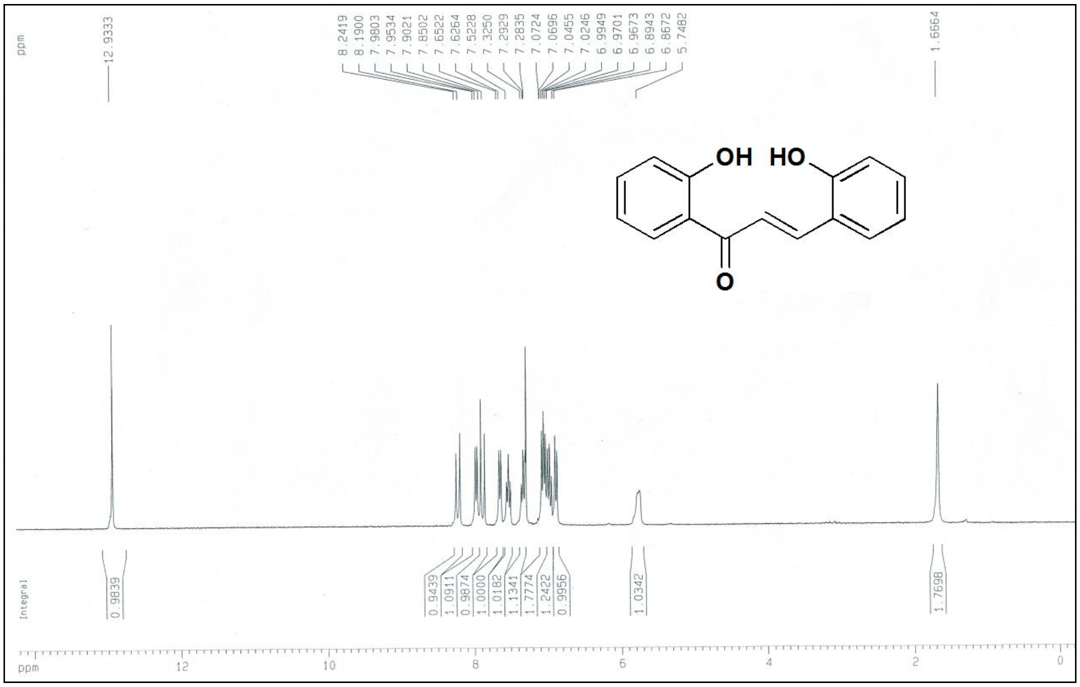

Fig. 7: ^{1}H NMR Spectrum of 4 (300 MHz, $CDCl_3$)

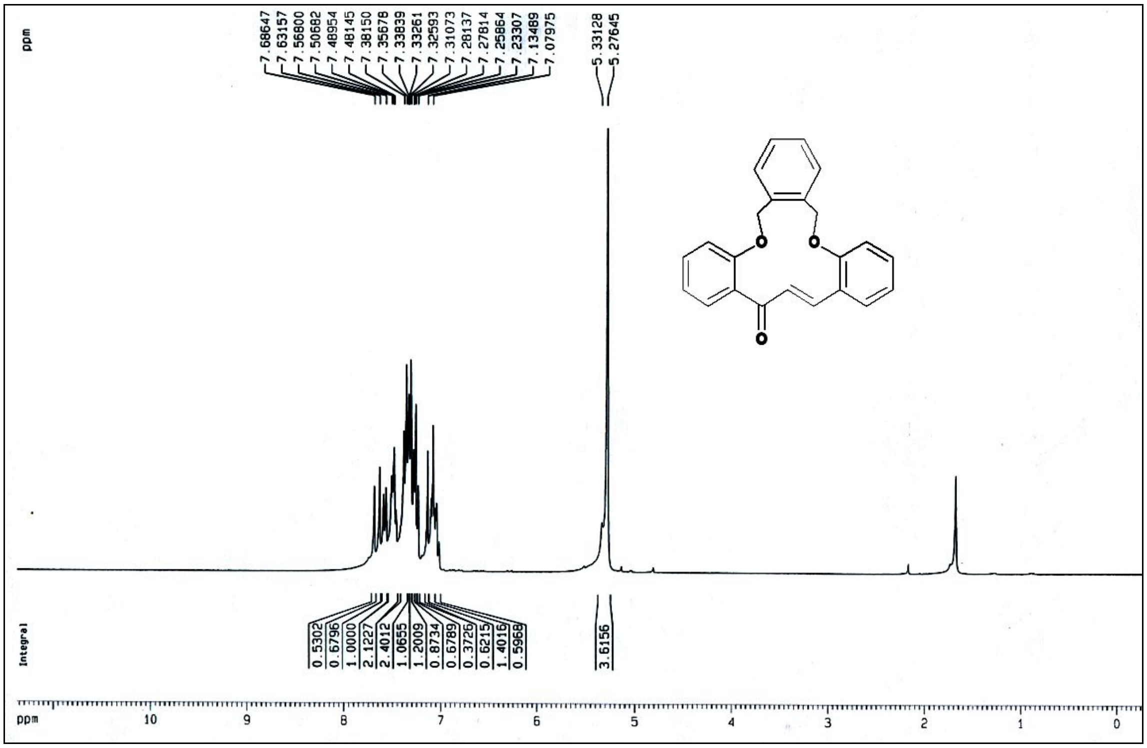

Fig. 8: ^{1}H NMR Spectrum of 5 (300 MHz, $CDCl_3$)

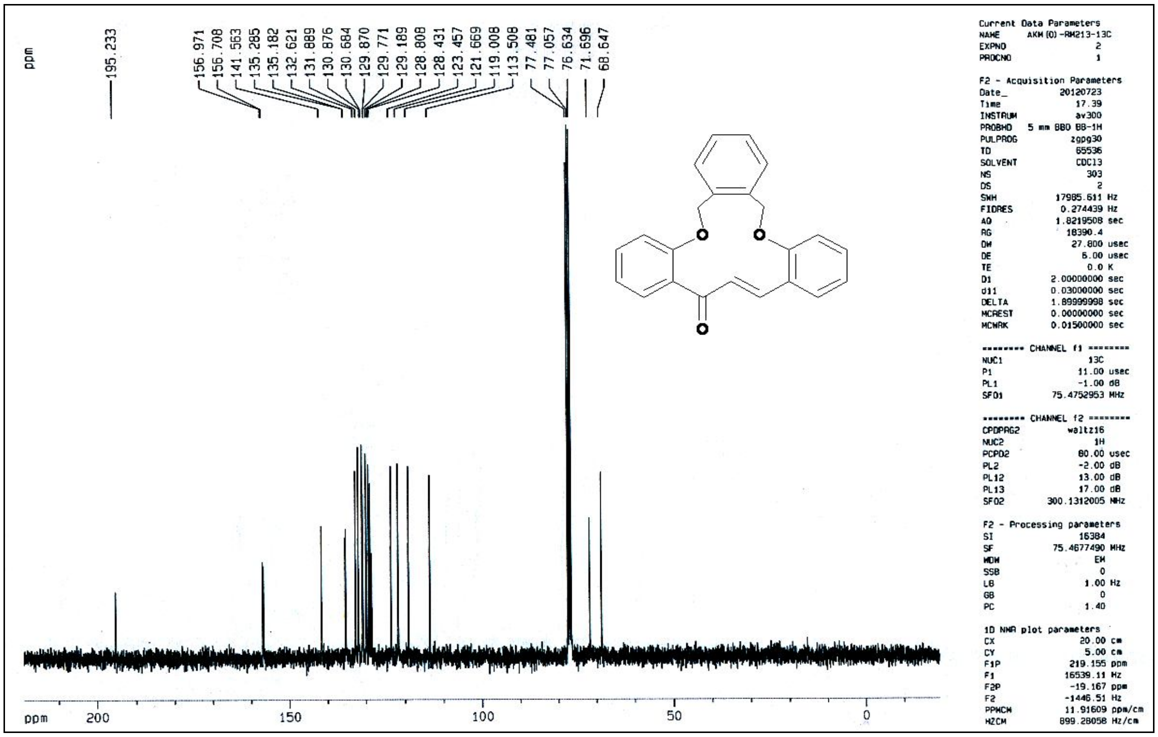

Fig. 9: ^{13}C NMR Spectrum of 5 (75 MHz, $CDCl_3$)

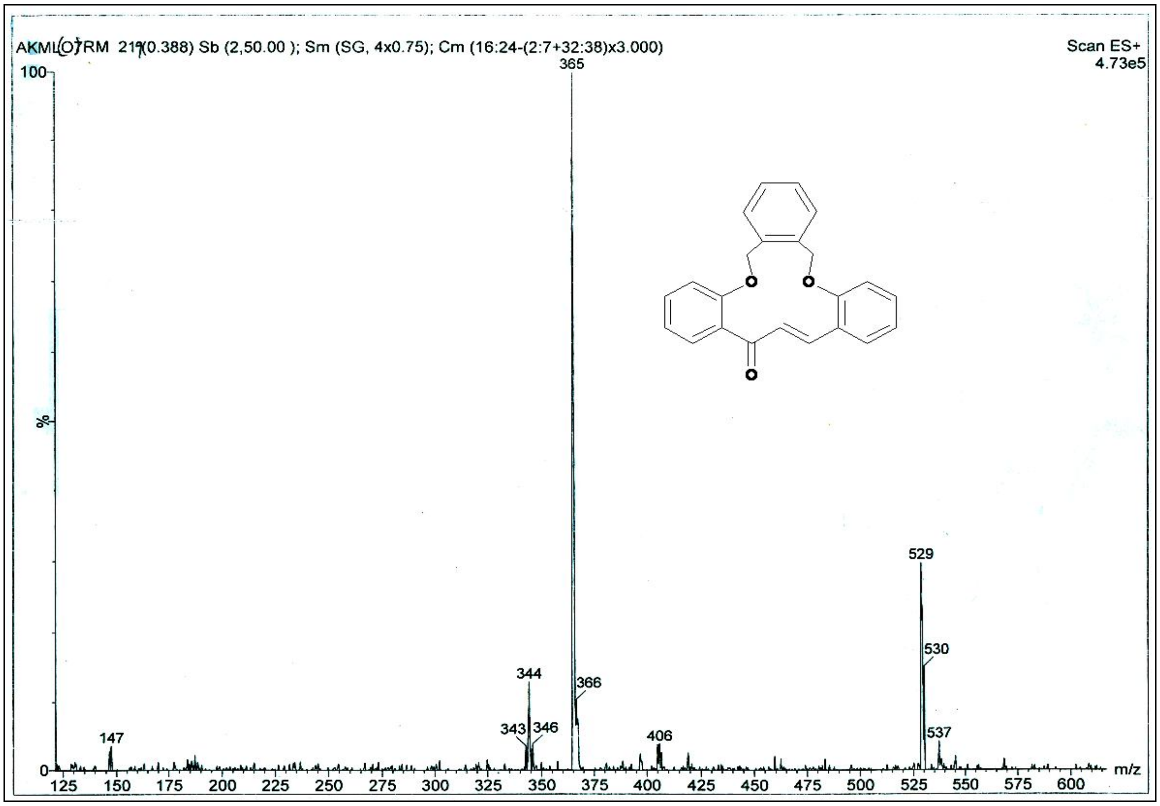

Fig. 10: TOF MS ES+ Spectrum of 5

(19*E*,43*E*)-2.11.27.36-Tetroxaheptacyclo[44.4.0.0^{4,9}.0^{12,17}.0^{21,26}.0^{29,34}.0^{37,42}]pentaconta-1(46),4(9),5,7,12(17),13,15,19,21,23,25,29,31,33,37,39,41,43,47,49-icosaene-18,45-dione **(3)**:

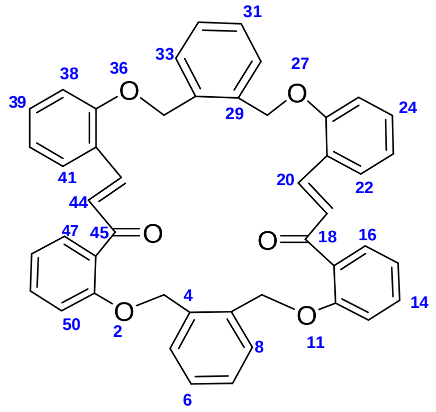

(19*E*)-2.11-Dioxatetracyclo[19.4.0.0^{4,9}.0^{12,17}]pentacosa-1(25),4(9),5,7,12(17),13,15,19,21,23-decaen-18-one **(5):**

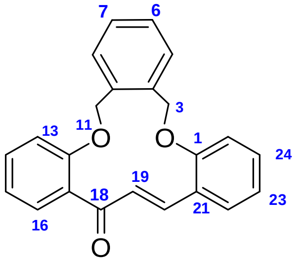

Supplement: Supplementary file 1 — Supplementary Material: Two tables containing the analytical and spectral data of 3 and 5 and ten figures showing the spectra of different compounds have been given. [file 485014.f1.pdf]
